# Synthesis of Esters of Ginsenoside Metabolite M1 and Their Cytotoxicity on MGC80-3 Cells

**DOI:** 10.3390/molecules18043689

**Published:** 2013-03-25

**Authors:** Wen-Fang Li, Li-Rong Chen, Xiao-Jie Gong, Zheng-Ning Li, Ke-Ke Li

**Affiliations:** 1 College of Medical, Dalian University, Dalian 116622, China; 2 College of Environmental and Chemical Engineering, Dalian University, Dalian 116622, China

**Keywords:** ginsenoside, synthesis, cytotoxicity, MGC80-3

## Abstract

Monoesters of ginsenoside metabolite M1 at the 3-OH, 4-OH and 6-OH positions of the glucose moiety at M1 were synthesized via the reaction of M1 with acyl chloride, or acid-*N,N'*-diisopropylcarbodiimide in the presence of DMAP. Their structures were fully characterized by spectral methods. The cytotoxicity of these compounds against then MGC80-3 human gastric cancer cell line was also assessed. High inhibitory effects were found at a concentration of 100 μg/mL.

## 1. Introduction

**Figure 1:**
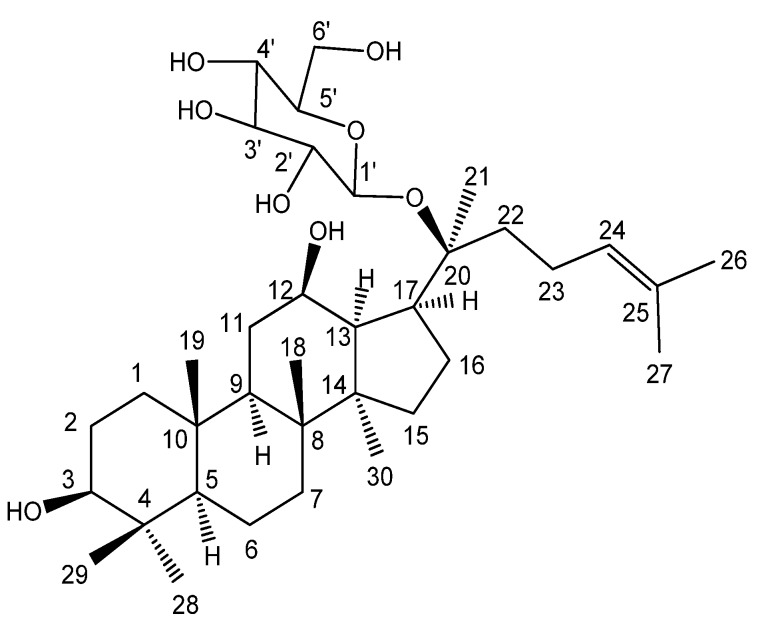
The structure and atom numbering of M1.

Ginseng (the roots of *Panax ginseng* C. A. MEYER. Araliaceae) has long been used in eastern Asia for their medicinal effects. The beneficial effects of ginseng are attributed to the bioactive components presenting in it, and lead to the isolation and identification of the ginsenosides, which are glycosides containing an aglycone (protopanaxadiol or protopanaxatriol) with a dammarane skeleton. On the other hand, the effect might rise from the metabolites of the glycosides when absorbed by human beings. Pharmaceutical studies have shown that upon oral administration of ginseng extract or ginsenosides they are deglycosylated by intestinal bacteria into the ginseng saponin metabolite 20-*O*-*β*-D-glucopyranosyl-20(*S*)-protopanaxadiol (M1, Figure 1), as the main metabolite [1,2,3,4,5,6,7,8,9,10,11]. M1 displays anticancer activity through the induction of apoptosis in various types of cancer cells [12,13,14,15,16]. Wang proposed that it is M1, instead of Rb_1_ (a primary glycoside in *P. ginseng*), that possesses potentially chemopreventive activities in human colorectal cancer [17]. Recently, Kim *et al*. reported that M1 could be used to treat inflammatory diseases, such as colitis, by targeting IRAK-1 activation as it inhibited the production of proinflammatory cytokines more potently than those of ginsenoside Rb_1_ did [18]. Other beneficial metabolic effects, e.g., enhancement of insulin secretion using M1 [19], and the anti-diabetic activity of M1 [20], have also been reported. A very recent report revealed that M1 inhibited the expressions of inducible nitric-oxide synthase, proinflammatory cytokines, monocyte chemotactic protein-1, matrix metalloproteinase-3, and matrix metalloproteinase-9 in lipopolysaccharide (LPS)-stimulated BV2 microglial cells [21]. It suppresses microglial activation by inhibiting reactive oxygen species, and thus, is a promising agent for the treatment of various neurologic disorders. Accordingly, it is not surprising that the transformation of ginseng or notoginseng saponins (from the herb *P. notoginseng*, which chemical constituents are similar to the ginsenosides) into M1 and the bioactivities of M1 have been the subject of intense focus in the last decade [22,23,24,25].

Hasegawa proposed that the metabolized M1 was esterified with fatty acids in the liver, which resulted in longer permanence in the body and exhibits the antitumor activities, and concluded that ginsenoside is a pro-drug activated in the body by deglycosylation and esterification [2,11]. Compared to the fact that much attention has been devoted to the bioactivities, especially anticancer activity of M1 [15,16,17], there is scant synthetic work related to M1 in the literature [26,27]. Recently, we synthesized the monoesters of M1 via introduction of acyl groups onto the 6'-OH of the glucose moiety in M1 and found the esterified products exhibited cytotoxicity against several human cancer cell lines (breast cancer MCF-7, skin melanoma SK-MEL-2 and human ovarian carcinoma B16) [28].

**Figure 2:**
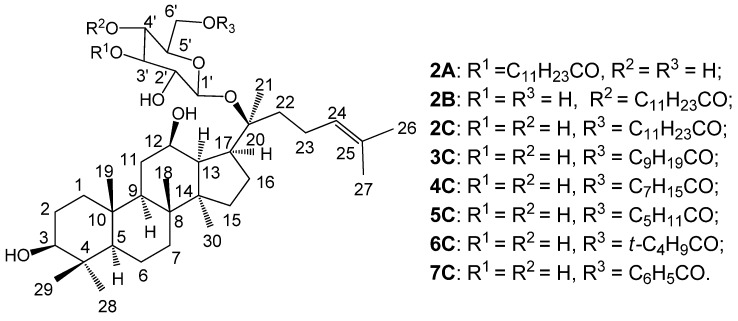
The structures of M1 esters **2**–**7**.

Even though it is reported that only the fatty acid ester substituent was connected to the C-6' position [10], there is no experimental evidence to prove it. On the other hand, it is imaginable that there may be several possible esterified products due to the existence of multiple hydroxyl groups in M1, each being possibly esterified. These isomeric esters may display different bioactivities. A systemic investigation of the anti-tumor activity should be important for understanding the metabolite products and the possible discovery of more active anti-tumor compounds. Meanwhile, the existence of six hydroxyls in M1 makes the esterification products very complex, even for the monoacylation products, and makes the synthesis and separation challenging. There are few reports about chemical esterification of M1, even about directed and selective esterification of glycosides [29]. We tried to synthesize the M1 esters by introducing acyl groups onto one of the OH’s in M1. Herein, we wish to report that the the 3'-OH, or 4'-OH or 6'-OH of the glucose moiety in M1 can be monoesterified, and three different kinds of monoesters of M1 could be thus obtained (Figure 2). In addition, some of their antitumor activities are also reported.

## 2. Results and Discussion

### 2.1. Synthesis

First, some new esters were synthesized from ginsenoside M1 and carboxylic acids, in the presence of promoting reagents. Due to presence of multiple hydroxyl groups in M1, it is a great challenge to carry out the esterification selectively, even to obtain a mixture of monoesterified products. Introduction of a lauroyl (dodecanoyl) to M1 was performed using the acid-*N,N'*-diisopropylcarbodiimide (DIC) or the acyl chloride as the acylation reagents. Multiple-spots were found in TLC monitoring. Three monoesters of M1 were isolated, and they are assigned as the esters bearing an acyloxy group at the C-3, C-4, C-6 positions in the glucose moiety of M1, respectively (see Section 2.2). Among the three monoesters, the C-6 ester is the most polar one. TLC spots corresponding to the three monoesters were found in all cases. Besides the monoesters, there are also less polar products formed, which might be di- or multi-esterified products of M1, but the attempts to separate them by column chromatography were unsuccessful. As shown in Table 1, a 54% overall yield of the monoesters was obtained using 100 mol% of the acyl chloride as the acylating agent, Et_3_N as the solvent and base and DMAP as the catalyst. In this way, the reaction is fast and the yield relatively high. Even though 3-[*N*-decyl-*N*-(4-pyridyl)amino]propionic acid (DPAP) has been reported to be a catalyst favoring the selective introduction of acyl group to the 6'-OH of the glucose [30], it was not successful for the reaction of M1 with dodecanoic acid (entry 4).

**Table 1:** The optimization of reaction conditions.

Entry	Acylating reagent	Cat.	M1: Acylating reagent: Cat. molar ratio	Time (h)	Yield 2C (%)
1	C_11_H_23_CO_2_H-DIC	DMAP	1:1:0.5	6	12.5
2	C_11_H_23_CO_2_H-DIC	DMAP	0.5:1:0.3	6	4.6
3	C_11_H_23_CO_2_H-DIC	DMAP	1:2:1	9	5.1
4	C_11_H_23_CO_2_H-DIC	DPAP	1:1:0.5	6	8.7
5	C_11_H_23_COCl	DMAP	1:1:0.5	0.5	54.1

Dichloromethane as the solvent, RT.

With the optimal reaction conditions in hand, reactions of M1 with other carboxylic acid chlorides were carried out, and the results are listed in Table 2. Yields in the 41–54% range were obtained for the synthesis of esters of aromatic acid and aliphatic acids.

**Table 2:** Synthesis of the monoesters of M1 ^a^.

Entry	RCOCl	Time (h)	Prod.	Yield (%)
1	C_11_H_23_COCl	0.6	**2C**	48 ^b^
2	C_9_H_19_COCl	0.5	**3C**	43 ^b^
3	C_7_H_15_COCl	0.5	**4C**	48 ^b^
4	C_5_H_11_COCl	0.5	**5C**	41 ^b^
5	*t*-C_4_H_9_COCl	2.0	**6C**	48 ^b^
6	C_6_H_5_COCl	0.5	**7C**	54 ^c^

^a^ Molar ratio of M1/RCOCl/DMAP = 1:1:0.02; ^b^ isolated yield unless otherwise noted; ^c^ the yield by HPLC.

### 2.2. Structure Characterization of the New Monoesters ***2A*** and ***2B***

Previously, we reported the C-6' ester of M1 [28]. Since no characterization data could be found for the two new M1 monoesters, we recorded the MS, IR and NMR spectral data, and the structures of the products were deduced from these data. The monoesters are coded as **2A**, **2B**, and **2C** according to the relative polarity in TLC from the weakest to the strongest.

The strong IR absorptions for **2A**, **2B** and **2C** at 1730, 1743, 1730 cm^−1^, respectively, clearly indicated the presence of ester groups in these compounds, along with the absorptions at 3369–3389 cm^−1^ due to the existence of hydroxy group(s). In high-resolution positive-mode FAB-MS (*m/z*), major signals at 827.6005, 827.5996, 827.5687 for **2A**, **2B** and **2C**, respectively, are in agreement with the composition [C_48_H_84_O_9_Na]^+^, therefore, **2A**, **2B** and **2C** all have the same composition of C_48_H_84_O_9_, which corresponds to the monolauroyl ester of M1.

**Figure 3:**
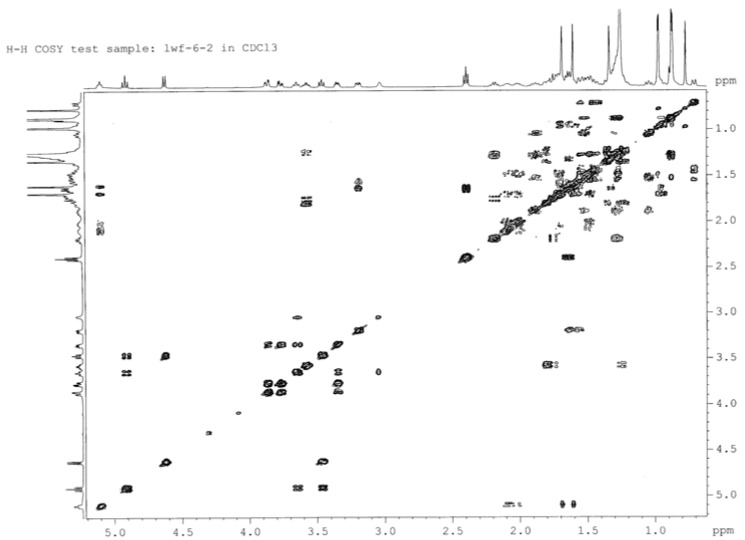
^1^H-^1^H COSY spectrum of **2A**.

**Figure 4:**
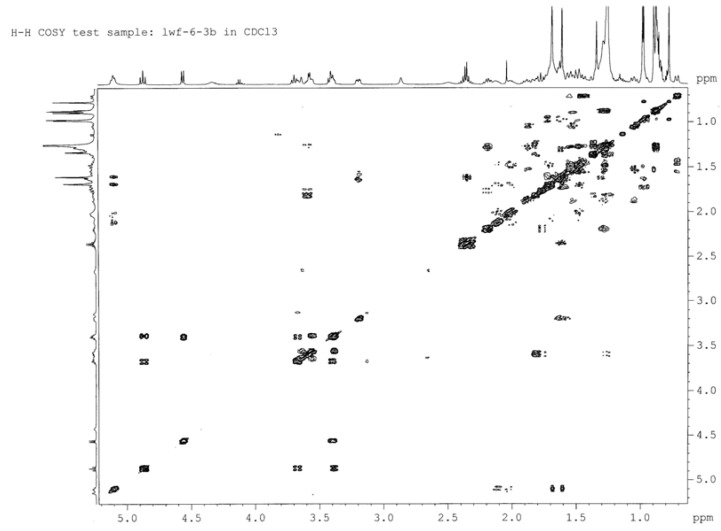
^1^H-^1^H COSY spectrum of **2B**.

**Figure 5:**
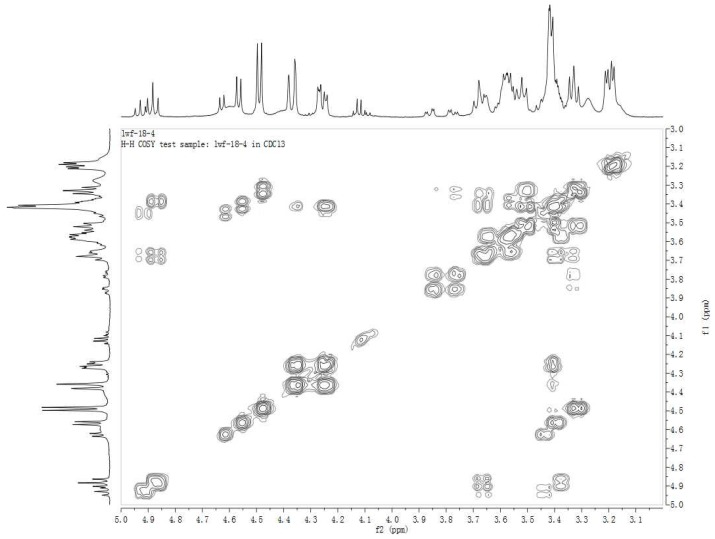
^1^H-^1^H COSY spectrum of **2C** (part).

**Figure 6:**
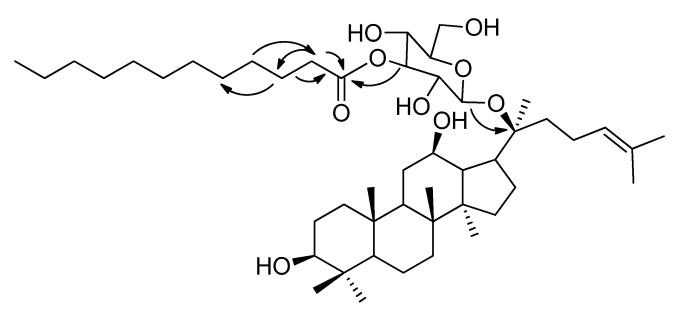
Selected key HMBC correlations of **2A** (H→C).

The connection positions of the acyl to M1 were deduced from NMR data. First, the ^1^H-NMR signals in the range of *δ* = 5.2–3.0 ppm for **2A**, **2B** and **2C** were assigned from the ^1^H-^1^H COSY (Figure 3, Figure 4, Figure 5), starting from the dual proton peak attached to C-1'. The combination of ^1^H-^1^H COSY, DEPT, and HSQC leads to the assignment of most of the signals. Then, an unambiguous structure assignment of **2A**, **2B** and **2C** was achieved with resort to the HMBC data (Figure 6), where the correlations between the ^13^C signal of the carbonyl and proton attached to the glucose moiety were observed. Thus, **2A**, **2B** and **2C** were confirmed as monoesters of M1 which contain a lauroyloxy group at the C-3', C-4', and C-6' positions, respectively. Even though no much difference is seen in the NMR for the aglycone moiety in M1 and its esters, the signals corresponding to the glycosyl are very different. The ^1^H-NMR and ^13^C-NMR data for the glucose moiety in M1, **2A**, **2B** and **2C** are listed in Table 3, Table 4, respectively. The introduction of an acyl group to the hydroxy group in the glucose moiety results in a remarkable downfield shift of the proton signal attached to the carbon which connects an acyloxy group. In **2A**, the signal for the proton at C-3' appeared at *δ* 4.91 ppm, which is ∆*δ* 1.43 ppm downfield when compared to that in M1. The H-4' signal in **2B** changes to *δ* 4.87 ppm, whereas the corresponding H-4' signal in M1 is at *δ* 3.59 ppm. For **2C**, there is a ∆*δ* 0.48 ppm downfield shift for H-6'. In the ^13^C-NMR spectra of **2A** and **2C**, there are 1.33–1.84 ppm changes to high-field for the carbons connecting an acyl group. In **2B**, the C-4' signal is abnormally shifted to downfield by 0.45 ppm. In **2A**, **2B** and **2C**, the NMR signals of the carbons proximal to the carbon attached to an acyloxyl group appear remarkably downfield, in the range of *δ* 1.29–2.49 ppm. Therefore, it would be easy to judge the connecting mode of a monoester of M1. Trends of the change of chemical shifts in both ^1^H-NMR and ^13^C-NMR, except that of the C-4' signal in **2B**, are in agreement with the literature data [31,32].

### 2.3. Cytotoxicity of the Synthesized Monoesters

The monoesters of M1 were subjected to bioassays against the human gastric cancer cell line MGC80-3, and the results are shown in Table 5. All the esters derived from M1 show strong cytotoxic activities against MGC80-3 cells at 24 h and 48 h at a concentration of 100 μg/mL. The cytotoxicity is much higher than in the control experiment using 5-FU at 10 μg/mL. Even though the mass concentrations of the M1 esters are one order higher than that of 5-FU, their molar concentrations are very close each other. M1 esters with a longer chain acyl displayed higher activities than those with shorter chains against the cells. The M1 ester of benzoic acid is also highly effective at inhibiting the cells. Remarkably, **2B**, 4'-C_11_CO_2_-M1, at 10 μg/mL can inhibit 43% of the cells in 24 h.

**Table 3:** ^1^H-NMR data of the glycosyls in M1, **2A**, **2B** and **2C** in CDCl_3_ (*δ* in ppm, *J* in Hz, recorded at 500 MHz).

Position	*δ*_M1_	*δ*_2A_ (C-3' ester)	*δ*_2B_ (C-4' ester)	*δ*_2C_ (C-6' ester)
1'	4.57 (d, 7.3)	4.62 (d, 7.8)	4.56 (d, 7.7)	4.49 (d, 7.7)
2'	3.21 (dd, 8.0)	3.46 (t, 8.9)	3.41 (t, 8.4)	3.31 (m)
3'	3.46 (m)	4.91 (t, 9.3)	3.69 (t, 9.4)	3.42 (m)
4'	3.61 (m)	3.65 (t, 9.1)	4.87 (t, 9.6)	3.62 (m)
5'	3.33 (m)	3.35 (m)	3.38 (m)	3.35 (m)
6'	3.77 (dd, 7.5, 11.2)	3.87 (dd, 3.3, 11.9) 3.77 (dd, 4.7, 11.9)	3.65 (m) 3.56 (m)	4.24 (dd, 11.0, 5.4) 4.37 (d, 11.2)

**Table 4:** ^13^C-NMR data of the glycosyls in M1, **2A**, **2B** and **2C** in CDCl_3_ (*δ* in ppm, *J* in Hz, recorded at 125 MHz).

Position	*δ*_M1_	*δ*_2A_	*δ*_2A_-*δ*_M1_	*δ*_2B_	*δ*_2B_-*δ*_M1_	*δ*_2C_	*δ*_2C_-*δ*_M1_
1'	98.0	97.3	−0.7	96.8	−1.2	97.0	−1.0
2'	73.7	72.0	−1.7	74.3	0.6	73.4	−0.3
3'	77.0	78.3	1.3	74.7	−2.3	76.8	−0.2
4'	70.1	69.6	−0.5	70.5	−0.4	70.1	0.0
5'	76.0	75.6	−0.4	74.1	−1.9	73.6	−2.4
6'	61.5	62.4	0.9	61.7	0.2	63.2	1.7

**Table 5:** Cytotoxic efffects of monoesters of M1 on MGC80-3 ^a^.

Samples	Inhibitory in 24 h (%) (%)	Inhibitory in 48 h (%)
**2B**	45.9	98.1
**3C**	97.7	78.9
**4C**	53.1	54.6
**5C**	32.4	86.4
**6C**	42.4	57.7
**7C**	82.0	90.4
5-FU ^b^	14.8	37.0

^a^ 100 μg/mL unless noted; ^b^ 10 μg/mL.

## 3. Experimental

### 3.1. General

All chemicals were purchased from Alfa Aesar Co., Ltd. (Tianjin, China) except M1, which was provided by Dalian Polytechnic University. NMR spectra were recorded on a Bruker DRX500 spectrometer in CDCl_3_ using TMS as an internal standard. IR spectra were recorded on a Nicolet 550 spectrometer. HRMS were recorded on a Micromass UPLC/Q-TOF Micro spectrometer.

### 3.2. Synthesis of Monoesters of M1

To a solution of M1 (100 mg) in CH_2_Cl_2_ (5.0 mL) were added DMAP (1.4 mg), C_11_H_23_COOH (67 mg) and *N,N'*-diisopropylcarbodiimide in sequence at 0 °C. The mixture was stirred at that temperature until TLC monitoring indicated most of M1 was consumed. After quenching the reaction by adding aqueous Na_2_CO_3_, extraction, drying and evaporation of the solvent, the residue was purified by column chromatography on silica gel. Elution with EA/PE (1:4 to 3:7 to 1:1) gave **2A**, **2B** and **2C**.

*3β,12β,20(S)-Trihydroxy-dammar-24-ene 20-O-β-D-glucopyranoside* (**M1**). ^1^H-NMR (500 MHz, CDCl_3_) *δ* 5.07 (t, *J* = 6.4 Hz, 1H, H-24), 4.57 (d, *J* = 7.3 Hz, 1H, H-1'), 3.77 (dd, *J* = 19.0, 11.3 Hz, 2H, H-6'), 3.61 (m, 1H, H-4'), 3.58 (s, br, 2H), 3.53 (m, 1H, H-12), 3.46 (m, 1H, H-3'), 3.33 (m, 1H, H-5'), 3.23 (m, 1H, H-3), 3.21 (d, *J* = 8.0 Hz, 1H, H-2'), 2.18 (dd, *J* = 17.6, 8.8 Hz, 1H, H-17), 2.03 (m, 2H, H-23), 1.87 (m, 2H, H-16), 1.85–1.71 (m, 5H), 1.69 (s, 3H, H-26), 1.64 (m, 2H, H-22), 1.60 (s, 3H, H-27), 1.52 (m, 2H, H-15), 1.54–1.31 (m, 10H), 1.30 (s, 3H, H-21), 1.22 (m, 1H, Ha-7), 0.97 (s, 6H, H-19, H-28), 0.90 (s, 3H, H-30), 0.87 (s, 3H, H-18), 0.77 (s, 3H, H-29), 0.72 (d, *J* = 10.1 Hz, 1H, H-5); ^13^C-NMR (125 MHz, CDCl_3_) *δ* 131.7 (C-25), 124.7 (C-24), 98.0 (C-1'), 84.1 (C-20), 79.0 (C-3), 77.0 (C-3'), 76.0 (C-5'), 73.7 (C-2'), 70.1 (C-4'), 69.2 (C-12), 61.5 (C-6'), 56.1 (C-5), 52.2 (C-17), 51.7 (C-14), 50.1 (C-9), 48.4 (C-13), 39.9 (C-8), 39.2 (C-4), 39.1 (C-1), 37.2 (C-10), 35.4 (C-22), 34.9 (C-7), 30.9 (C-11), 30.2 (C-15), 28.2 (C-28), 27.5 (C-2), 26.7 (C-16), 25.9 (C-26), 23.2 (C-23), 22.5 (C-21), 18.4 (C-6), 18.0 (C-27), 17.3 (C-30), 16.3 (C-29), 15.9 (C-18), 15.6 (C-19).

*3β,12β,20(S)-Trihydroxy-dammar-24-ene 20-O-β-D-glucopyranosyl 3'-lauroyl ester* (**2A**). White powder. mp: 92–93 °C. [α]^25^_D_ + 25° (*c* 1.02, CH_2_Cl_2_). IR (KBr): 3362, 2966, 2932, 2851, 1730, 1629, 1454, 1387 cm^−1^. HRMS (*m/z*): 827.6005 [M+Na]^+^ (calcd. for C_48_H_84_O_9_Na: 827.6013). ^1^H-NMR (CDCl_3_, 500 MHz) *δ* 5.10 (t, *J* = 6.6 Hz, 1H, H-24), 4.91 (t, *J* = 9.3 Hz, 1H, H-3'), 4.62 (t, *J* = 7.8 Hz, 1H, H-1'), 3.87 (dd, *J* = 3.3 Hz, 1H, Ha-6'), 3.77 (dd, *J* = 4.7, 11.9 Hz, 1H, Hb-6'), 3.65 (t, *J* = 9.1 Hz, 1H, H-4'), 3.58 (dt, *J* = 5.3, 10.3 Hz, 1H, H-12), 3.46 (t, *J* = 8.9 Hz, 1H, H-2'), 3.35 (m, 1H, H-5'), 3.19 (dd, *J* = 4.6 Hz, 1H, H-3), 3.03 (s, br, 1H), 2.40 (t, *J* = 7.5 Hz, 2H, H-2''), 2.18 (m, 1H, H-17), 2.07 (m, 1H, Ha-23), 2.00 (m, 1H, Hb-23), 1.89 (m, 1H, Ha-16), 1.85–1.71 (m, 7H), 1.69 (s, 3H, H-26), 1.66–1.62 (m, 4H), 1.61 (s, 3H, H-27), 1.56–1.38 (m, 6H), 1.34 (s, 3H, H-21), 1.31–1.19 (m, 21H), 0.98 (s, 3H, H-28), 0.97 (s, 3H, H-19), 0.97 (m, 1H, Hb-1),0.89 (s, 3H, H-30), 0.88 (t, *J* = 7.3 Hz, 3H, H-12''), 0.87 (s, 3H, H-18), 0.78 (s, 3H, H-29), 0.71 (m, 1H, H-5); ^13^C-NMR (125 MHz, CDCl_3_) *δ* 175.6 (C-1''), 131.8 (C-25), 124.3 (C-24), 97.3 (C-1'), 84.6 (C-20), 78.9 (C-3), 78.3 (C-3'), 75.6 (C-5'), 72.0 (C-2'), 70.6 (C-12), 69.6 (C-4'), 62.4 (C-6'), 55.9 (C-5), 51.7 (C-17), 51.5 (C-14), 49.8 (C-9), 48.1 (C-13), 39.8 (C-8), 39.0 (C-4), 38.9 (C-1), 37.1 (C-10), 35.4 (C-22), 34.8 (C-7), 34.4 (C-2''), 31.9 (C-10''), 30.6 (C-11), 30.4 (C-15), 29.7 (C-7''), 29.6 (C-6''), 29.5 (C-8''), 29.4 (C-5''), 29.3 (C-9''), 29.1 (C-4''), 28.1 (C-28), 27.4 (C-2), 26.6 (C-16), 25.7 (C-26), 25.3 (C-3''), 24.9 (C-23), 22.7 (C-11''), 22.4 (C-21), 18.3 (C-6), 17.8 (C-27), 17.0 (C-30), 16.1 (C-29), 15.8 (C-18), 15.4 (C-19), 14.1 (C-12'').

*3β,12β,20(S)-Trihydroxy-dammar-24-ene 20-O-β-D-glucopyranosyl 4'-lauroyl ester* (**2B**). Colorless oil. [α]^25^_D_ + 17° (*c* 2.00, CH_2_Cl_2_). IR (KBr): 3389, 2966, 2919, 2851, 1743, 1663, 1622, 1461, 1387, 1622 cm^−1^. HRMS (*m/z*): 827.5996 [M+Na]^+^ (calcd. for C_48_H_84_O_9_Na: 827.6013). ^1^H-NMR (500 MHz, CDCl_3_) *δ* 5.10 (s, br, 1H), 5.09 (t, *J* = 6.6 Hz, 1H, H-24), 4.87 (t, *J* = 9.6 Hz, 1H, H-4'), 4.56 (d, *J* = 7.7 Hz, 1H, H-1'), 4.33 (s, br, 2H), 3.69 (t, *J* = 9.6 Hz, 1H, H-3'), 3.65 (m, 1H, Ha-6'), 3.59 (m, 1H, H-12), 3.56 (m, 1H, Hb-6'), 3.41 (t, *J* = 8.4 Hz, 1H, H-2'), 3.38 (m, 1H, H-5'), 3.20 (dd, *J* = 11.2, 4.6 Hz, 1H, H-3), 2.86 (s, br, 1H), 2.37 (m, 2H, H-2''), 2.21 (m, 2H, H-23), 1.93–1.73 (m, 5H), 1.69 (s, 3H, H-26), 1.64 (m, 2H, H-22), 1.61 (s, 3H, H-27), 1.58–1.36 (m, 6H), 1.34 (s, 3H, H-21), 1.33–1.28 (m, 6H), 1.27–1.24 (m, 10H), 1.19–1.02 (m, 3H), 0.99 (s, 3H, H-28), 0.98 (s, 3H, H-19), 0.90 (s, 3H, H-30), 0.88 (s, 3H, H-18), 0.87 (m, 3H, H-12''), 0.87–0.82 (m, 6H), 0.78 (s, 3H, H-29), 0.72 (d, *J* = 11.4 Hz, 1H, H-5); ^13^C-NMR (125 MHz, CDCl_3_) *δ* 174.0 (C-1''), 131.9 (C-25), 124.3 (C-24), 96.8 (C-1'), 84.4 (C-20), 78.9 (C-3), 74.7 (C-3'), 74.3 (C-2'), 74.1 (C-5'), 70.7 (C-12), 70.5 (C-4'), 61.7 (C-6'), 55.9 (C-5), 51.7 (C-17), 51.5 (C-14), 49.9 (C-9), 48.1 (C-13), 39.8 (C-8), 39.0 (C-4), 38.9 (C-1), 37.1 (C-10), 35.5 (C-22), 34.8 (C-7), 34.4 (C-2''), 31.9 (C-10''), 30.7 (C-11), 30.5 (C-15), 29.7 (C-7''), 29.6 (C-6''), 29.5 (C-8''), 29.3 (C-5''), 29.2 (C-9''), 29.1 (C-4''), 28.1 (C-28), 27.4 (C-2), 26.7 (C-16), 25.7 (C-26), 25.3 (C-3''), 24.9 (C-23), 22.7 (C-11''), 22.4 (C-21), 18.3 (C-6), 17.8 (C-27), 17.0 (C-30), 16.2 (C-29), 15.8 (C-18), 15.4 (C-19), 14.1 (C-12'').

*3β,12β,20(S)-Trihydroxy-dammar-24-ene 20-O-β-D-glucopyranosyl 6'-lauroyl ester* (**2C**). Colorless oil. [α]^25^_D_ + 16° (*c* 1.01, CH_2_Cl_2_). IR (KBr): 3389, 2959, 2925, 2851, 1730, 1622, 1461, 1407 cm^−1^. HRMS (*m/z*): 827.5687 [M + Na]^+^ (calcd. for C_48_H_84_O_9_Na: 827.6013). ^1^H-NMR (500 MHz, CDCl_3_) *δ* 5.24 (s, br, 1H), 5.10 (t, *J* = 6.6 Hz, 1H, H-24), 4.49 (d, *J* = 7.7 Hz, 1H, H-1'), 4.30 (s, br, 2H), 4.37 (d, *J* = 11.2 Hz, 1H, Ha-6'), 4.24 (dd, *J* = 11.0, 5.4 Hz, 1H, Hb-6'), 3.67 (dd, *J* = 16.2, 6.9 Hz, 1H, H-12), 3.62 (m, 1H, H-4'), 3.42 (m, 1H, H-3'), 3.35 (m, 1H, H-5'), 3.31 (m, 1H, H-2'), 3.28 (s, br, 1H), 3.20 (dd, *J* = 11.3, 4.7 Hz, 1H, H-3), 2.31 (t, *J* = 7.6 Hz, 2H, H-2''), 2.19 (m, 2H, H-23), 1.95 (m, 1H, H-13), 1.91–1.73 (m, 8H), 1.68 (s, 3H, H-26), 1.63 (m, 2H, H-22), 1.60 (s, 3H, H-27), 1.58–1.37 (m, 8H), 1.34 (s, 3H, H-21), 1.31–1.21 (m, 18H), 1.02 (m, 1H, Ha-15), 0.98 (s, 3H, H-28), 0.97 (s, 3H, H-19), 0.90 (s, 3H, H-30), 0.88 (s, 3H, H-18), 0.87 (t, *J* = 7.2 Hz, 3H, H-12''), 0.78 (s, 3H, H-29), 0.72 (d, *J* = 11.3 Hz, 1H, H-5); ^13^C-NMR (125 MHz, CDCl_3_) *δ* 174.1 (C-1''), 131.6 (C-25), 124.6 (C-24), 97.0 (C-1'), 84.4 (C-20), 78.9 (C-3), 76.8 (C-3'), 73.6 (C-5'), 73.4 (C-2'), 70.7 (C-12), 70.1 (C-4'), 63.2 (C-6'), 55.9 (C-5), 51.8 (C-17), 51.4 (C-14), 49.8 (C-9), 47.9 (C-13), 39.8 (C-8), 39.0 (C-4), 38.9 (C-1), 37.1 (C-10), 35.5 (C-22), 34.8 (C-7), 34.2 (C-2''), 31.9 (C-10''), 30.6 (C-11), 30.3 (C-15), 29.7 (C-7''), 29.6 (C-6''), 29.5 (C-8''), 29.4 (C-5''), 29.3 (C-9''), 29.2 (C-4''), 28.1 (C-28), 27.4 (C-2), 26.8 (C-16), 25.7 (C-26), 25.3 (C-3''), 24.9 (C-23), 22.7 (C-11''), 22.1 (C-21), 18.3 (C-6), 17.7 (C-27), 17.0 (C-30), 16.2 (C-29), 15.8 (C-18), 15.4 (C-19), 14.1 (C-12'').

*3β,12β,20(S)-Trihydroxy-dammar-24-ene 20-O-β-D-glucopyranosyl 6'-decanoyl ester* (**3C**). Colorless oil. HRMS (*m/z*): 799.5667 [M + Na]^+^ (calcd. for C_46_H_80_O_9_Na: 799.5700). ^1^H-NMR (500 MHz, CDCl_3_) *δ* 5.24 (s, br, 1H), 5.11 (t, *J* = 7.0 Hz, 1H, H-24), 4.58 (s, br, 1H), 4.49 (d, *J* = 7.7 Hz, 1H, H-1'), 4.36 (d, *J* = 11.4 Hz, 1H, Ha-6'), 4.28 (dd, *J* = 11.3, 4.6 Hz, 1H, Hb-6'), 3.62 (m, 1H, H-12), 3.59 (m, 1H, H-4'), 3.42 (d, *J* = 5.5 Hz, 1H, H-3'), 3.34 (m, 1H, H-5'), 3.26 (m, 1H, H-2'), 3.20 (dd, *J* = 11.4, 4.8 Hz, 1H, H-3), 3.08 (s, br, 1H), 2.31 (t, *J* = 7.5 Hz, 2H, H-2''), 2.22 (m, 1H, H-17), 2.10 (m, 1H, Ha-23), 1.99 (m, 1H, H-13), 1.89 (m, 1H, Ha-16), 1.78 (m, 2H, H-2), 1.69 (s, 3H, H-26), 1.67–1.62 (m, 6H), 1.60 (s, 3H, H-27), 1.58–1.37 (m, 8H), 1.35 (s, 3H, H-21), 1.31–1.26 (m, 16H), 1.05 (m, 1H, Ha-15), 0.98 (s, 3H, H-28), 0.97 (s, 3H, H-19), 0.90 (s, 3H, H-30), 0.88 (s, 3H, H-18), 0.87 (t, *J* = 7.0 Hz, 3H, H-10''), 0.78 (s, 3H, H-29), 0.72 (d, *J* = 11.3 Hz, 1H, H-5); ^13^C-NMR (125 MHz, CDCl_3_) *δ* 174.2 (C-1''), 131.7 (C-25), 124.7 (C-24), 97.1 (C-1'), 84.5 (C-20), 79.0 (C-3), 76.8 (C-3'), 73.7 (C-5'), 73.6 (C-2'), 70.8 (C-12), 70.2 (C-4'), 63.5 (C-6'), 56.0 (C-5), 51.9 (C-17), 51.5 (C-14), 50.0 (C-9), 48.1 (C-13), 40.0 (C-8), 39.1 (C-4), 38.8 (C-1), 37.3 (C-10), 35.6 (C-22), 34.9 (C-7), 34.4 (C-2''), 32.0 (C-8''), 30.8 (C-11), 30.7 (C-15), 29.6 (C-6''), 29.4 (C-5'', C-7''), 29.3 (C-4''), 28.2 (C-28), 27.6 (C-2), 26.9 (C-16), 25.8 (C-26), 25.0 (C-3''), 24.9 (C-23), 22.8 (C-9''), 22.2 (C-21), 18.4 (C-6), 17.8 (C-27), 17.1 (C-30), 16.3 (C-29), 15.9 (C-18), 15.5 (C-19), 14.2 (C-10'').

*3β,12β,20(S)-Trihydroxy-dammar-24-ene 20-O-β-D-glucopyranosyl 6'-octanoyl ester* (**4C**). Colorless oil. HRMS (*m/z*): 771.5388 [M + Na]^+^ (calcd. for C_44_H_76_O_9_Na: 771.5387). ^1^H-NMR (500 MHz, CDCl_3_) *δ* 5.28 (s, br, 1H), 5.10 (t, *J* = 7.0 Hz, 1H, H-24), 4.69 (s, br, 1H), 4.50 (d, *J* = 7.7 Hz, 1H, H-1'), 4.39 (d, *J* = 10.5 Hz, 1H, Ha-6'), 4.22 (dd, *J* = 11.8, 5.8 Hz, 1H, Hb-6'), 3.72 (s, br, 1H), 3.59 (m, 1H, H-4'), 3.44 (m, 1H, H-3'), 3.36 (m, 1H, H-5'), 3.33 (t, *J* = 8.3 Hz, 1H, H-2'), 3.20 (dd, *J* = 11.1, 4.7 Hz, 1H, H-3), 2.30 (t, *J* = 7.6 Hz, 2H, H-2''), 2.14 (m, 1H, Ha-23), 2.08 (m, 1H, Ha-22), 2.02–1.70 (m, 8H), 1.68 (s, 3H, H-26), 1.66–1.62 (m, 4H), 1.60 (s, 3H, H-27), 1.57–1.43 (m, 4H), 1.40 (m, 2H, H-6), 1.36 (m, 1H, Ha-11), 1.34 (s, 3H, H-21), 1.32–1.24 (m, 12H), 1.02 (m, 1H, Ha-15), 0.98 (s, 6H, H-19, H-28), 0.89 (s, 3H, H-30), 0.87 (s, 3H, H-18), 0.85 (t, *J* = 6.8 Hz, 3H, H-8''), 0.78 (s, 3H, H-29), 0.72 (d, *J* = 11.2 Hz, 1H, H-5); ^13^C-NMR (125 MHz, CDCl3) *δ* 174.1 (C-1''), 131.5 (C-25), 124.8 (C-24), 96.9 (C-1'), 84.3 (C-20), 79.0 (C-3), 77.1 (C-3'), 73.7 (C-5'), 73.5 (C-2'), 70.7 (C-12), 70.3 (C-4'), 63.6 (C-6'), 56.1 (C-5), 51.8 (C-17), 51.5 (C-14), 50.0 (C-9), 48.1 (C-13), 39.9 (C-8), 39.0 (C-1, C-4), 37.2 (C-10), 35.6 (C-22), 34.9 (C-7), 34.3 (C-2''), 31.8 (C-6''), 30.8 (C-11), 30.5 (C-15), 29.3 (C-4''), 29.1 (C-5''), 28.2 (C-28), 27.5 (C-2), 26.8 (C-16), 25.8 (C-26), 25.1 (C-3''), 24.6 (C-23), 22.7 (C-7''), 22.3 (C-21), 18.4 (C-6), 17.8 (C-27), 17.1 (C-30), 16.3 (C-29), 15.9 (C-18), 15.5 (C-19), 14.2 (C-8'').

*3β,12β,20(S)-Trihydroxy-dammar-24-ene 20-O-β-D-glucopyranosyl 6'-hexyl ester* (**5C**). Colorless oil. HRMS (*m/z*): 743.5054 [M + Na]^+^ (calcd. for C_42_H_72_O_9_Na: 743.5074). ^1^H-NMR (500 MHz, CDCl_3_) *δ* 5.21 (s, br, 1H), 5.10 (t, *J* = 7.0 Hz, 1H, H-24), 4.53 (s, br, 1H), 4.52 (d, *J* = 7.7 Hz, 1H, H-1'), 4.38 (m, 1H, Ha-6'), 4.21 (dd, *J* = 11.8, 6.0 Hz, 1H, Hb-6'), 3.79 (s, br, 1H), 3.70 (m, 1H, H-12), 3.64 (m, 1H, H-4'), 3.45 (m, 1H, H-3'), 3.33 (t, *J* = 8.4 Hz, 1H, H-5'), 3.29 (m, 1H, H-2'), 3.20 (dd, *J* = 11.2, 4.8 Hz, 1H, H-3), 2.30 (t, *J* = 7.6 Hz, 2H, H-2''), 2.24–2.10 (m, 2H), 2.04–1.70 (m, 6H), 1.68 (s, 3H, H-26), 1.65–1.61 (m, 3H), 1.60 (s, 3H, H-27), 1.53–1.45 (m, 6H), 1.36 (m, 1H, Ha-11), 1.34 (s, 3H, H-21), 1.32–1.20 (m, 8H), 1.05 (m, 1H, Ha-15), 0.98 (s, 6H, H-19, H-28), 0.92–0.88 (m, 2H), 0.89 (s, 3H, H-30), 0.88 (t, *J* = 7.0 Hz, 3H, H-6''), 0.87 (s, 3H, H-18), 0.78 (s, 3H, H-29), 0.72 (d, *J* = 11.3 Hz, 1H, H-5); ^13^C-NMR (125 MHz, CDCl_3_) *δ* 174.0 (C-1''), 131.5 (C-25), 124.8 (C-24), 96.9 (C-1'), 84.2 (C-20), 78.9 (C-3), 77.1 (C-3'), 73.6 (C-5'), 73.5 (C-2'), 70.6 (C-12), 70.4 (C-4'), 63.7 (C-6'), 56.0 (C-5), 51.7 (C-17), 51.5 (C-14), 49.9 (C-9), 48.1 (C-13), 39.9 (C-8), 39.1 (C-1), 39.0 (C-4), 37.2 (C-10), 35.6 (C-22), 34.9 (C-7), 34.3 (C-2''), 31.4 (C-3'', C-4''), 30.7 (C-11), 30.5 (C-15), 28.2 (C-28), 27.5 (C-2), 26.7 (C-16), 25.8 (C-26), 24.7 (C-23), 22.4 (C-5''), 22.2 (C-21), 18.4 (C-6), 17.8 (C-27), 17.1 (C-30), 16.2 (C-29), 15.9 (C-18), 15.5 (C-19), 14.1 (C-6'').

*3β,12β,20(S)-Trihydroxy-dammar-24-ene 20-O-β-D-glucopyranosyl 6'-isobutyryl ester* **(6C**). Colorless oil. HRMS (*m/z*): 729.4910 [M + Na]^+^ (calcd. for C_41_H_70_O_9_Na: 729.4918). ^1^H-NMR (500 MHz, CDCl_3_) *δ* 5.25 (s, br, 1H), 5.10 (t, *J* = 7.0 Hz, 1H, H-24), 4.61 (s, br, 1H), 4.52 (d, *J* = 7.6 Hz, 1H, H-1'), 4.43 (dd, *J* = 11.7, 1.5 Hz, 1H, Ha-6'), 4.14 (dd, *J* = 11.9, 6.6 Hz, 1H, Hb-6'), 3.73 (s, br, 1H), 3.66 (m, 1H, H-12), 3.60 (m, 1H, H-4'), 3.45 (m, 1H, H-3'), 3.40 (t, *J* = 9.1 Hz, 1H, H-2'), 3.33 (t, *J* = 8.4 Hz, 1H, H-5'), 3.20 (dd, *J* = 11.3, 4.8 Hz, 1H, H-3), 2.23–2.15 (m, 3H), 1.92–1.78 (m, 3H), 1.78–1.69 (m, 2H), 1.68 (s, 3H, H-26), 1.66–1.61 (m, 4H), 1.59 (s, 3H, H-27), 1.55–1.46 (m, 4H), 1.37 (s, 3H, H-21), 1.32–1.21 (m, 6H), 1.19 (s, 9H, H-3'', H-4'', H-5''), 1.05 (m, 1H, Ha-15), 0.98 (s, 6H, H-19, H-28), 0.89 (s, 3H, H-30), 0.87 (s, 3H, H-18), 0.78 (s, 3H, H-29), 0.72 (d, *J* = 11.3 Hz, 1H, H-5); ^13^C-NMR (125 MHz, CDCl_3_) *δ* 178.7 (C-1''), 131.6 (C-25), 124.8 (C-24), 96.9 (C-1'), 84.3 (C-20), 79.0 (C-3), 77.0 (C-3'), 73.7 (C-5'), 73.6 (C-2'), 70.7 (C-12), 70.4 (C-4'), 64.0 (C-6'), 56.0 (C-5), 51.8 (C-17), 51.5 (C-14), 50.0 (C-9), 48.1 (C-13), 39.9 (C-8), 39.1 (C-1), 39.0 (C-4), 38.9 (C-2''), 37.2 (C-10), 35.6 (C-22), 34.9 (C-7), 30.8 (C-11), 30.6 (C-15), 28.2 (C-28), 27.5 (C-2), 27.3 (C-3'', C-4'', C-5''), 26.8 (C-16), 25.8 (C-26), 22.2 (C-23), 21.5 (C-21), 18.4 (C-6), 17.8 (C-27), 17.1 (C-30), 16.3 (C-29), 15.9 (C-18), 15.5 (C-19).

*3β,12β,20(S)-trihydroxy-dammar-24-ene 20-O-β-D-glucopyranosyl 6'-benzoyl ester* (**7C**). Colorless oil. HRMS (*m/z*): 749.4591 [M + Na]^+^ (calcd. for C_43_H_66_O_9_Na: 749.4605). ^1^H-NMR (500 MHz, CDCl_3_) *δ* 8.02 (d, *J* = 8.4 Hz, 2H, H-3'', H-7''), 7.54 (t, *J* = 7.4 Hz, 1H, H-5''), 7.41 (t, *J* = 7.8 Hz, 2H, H-4'', H-6''), 5.34 (s, br, 1H), 5.04 (t, *J* = 6.9 Hz, 1H, H-24), 4.87 (s, br, 1H), 4.67 (d, *J* = 10.1 Hz, 1H, Ha-6'), 4.54 (d, *J* = 7.7 Hz, 1H, H-1'), 4.46 (dd, *J* = 11.8, 6.9 Hz, 1H, Hb-6'), 4.23 (s, br, 1H), 3.87 (s, br, 1H), 3.64 (m, 1H, H-4'), 3.48 (t, *J* = 9.2 Hz, 1H, H-3'), 3.37 (t, *J* = 8.3 Hz, 1H, H-5'), 3.25 (m, 1H, H-2'), 3.16 (dd, *J* = 11.0, 5.0 Hz, 1H, H-3), 2.20–2.15 (m, 3H), 1.93–1.64 (m, 7H), 1.61 (s, 3H, H-26), 1.59–1.53 (m, 3H), 1.49 (s, 3H, H-27), 1.48–1.33 (m, 4H), 1.31 (s, 3H, H-21), 1.29–1.16 (m, 4H), 1.01 (m, 1H, Ha-15), 0.95 (s, 6H, H-19, H-28), 0.92 (m, 1H, Ha-1), 0.88 (s, 3H, H-30), 0.85 (s, 3H, H-18), 0.76 (s, 3H, H-29), 0.69 (d, *J* = 11.2 Hz, 1H, H-5); ^13^C-NMR (125 MHz, CDCl_3_) *δ* 166.7 (C-1''), 133.2 (C-5''), 131.5 (C-25), 130.1 (C-2''), 129.8 (C-3'', C-7''), 128.4 (C-4'', C-6''), 124.8 (C-24), 96.9 (C-1'), 84.3 (C-20), 79.0 (C-3), 77.2 (C-3'), 73.7 (C-5'), 73.5 (C-2'), 70.8 (C-12), 70.5 (C-4'), 64.4 (C-6'), 56.0 (C-5), 51.8 (C-17), 51.6 (C-14), 50.0 (C-9), 48.1 (C-13), 39.9 (C-8), 39.1 (C-1), 39.0 (C-4), 37.2 (C-10), 35.6 (C-22), 34.9 (C-7), 30.8 (C-11), 30.4 (C-15), 28.2 (C-28), 27.5 (C-2), 26.8 (C-16), 25.7 (C-26), 22.2 (C-23), 21.4 (C-21), 18.4 (C-6), 17.7 (C-27), 17.2 (C-30), 16.2 (C-29), 15.9 (C-18), 15.5 (C-19).

### 3.3. Bioassay

The 3-(4,5-dimethylthiazol-2-yl)-2,5-diphenyltetrazolium bromide (MTT) assay [12] was used to test the effect of M1 esters against MGC80-3 cells. MGC80-3 cells (Shanghai Life Academy of Sciences Cell Resource Center, Chinese Academy of Sciences, 200 μL) at a density of 5 × 10^3^/mL, 1 × 10^4^/mL, 2 × 10^4^/mL, 3 × 10^4^/mL, 4 × 10^4^/mL, 5 × 10^4^/mL, respectively, was added into the wells of 96-well microtiter plates. Each concentration was added to six wells, and three unvaccinated wells were used as the control. The plates were put in an incubation box (MCO-18AIC Sanyo Company, Beijing, China) with 5% CO_2_ at 37 °C for 24 h, 48 h and 72 h, respectively. Then, 20 µL of MTT solution (5 μg/mL) was added to each well and the plates were incubated for 4 h. 150 µL of DMSO was added to each well to dissolve the crystalline. The optical density (OD) values of the clear solutions obtained by centrifuge were measured using a microplate reader at 490 nm. The average data of six wells of the same conditions are presented as the percentage *versus* the blank experiment, which represents 100% cell viability.

## 4. Conclusions

In conclusion, we have synthesized a range of monoesters of M1, and identified their structures by spectral methods. An acyl group introduced to the 3'-OH, 4'-OH positions of M1 for the first time. These esters display anti-tumor activity towards the human gastric cancer cell line MGC80-3 at a concentration of 100 μg/mL.

## References

[1] Hasegawa H., Sung J.H., Matsumiya S., Uchiyama M. (1996). Main ginseng saponin metabolites formed by intestinal bacteria. Planta Med..

[2] Wakabayashi C., Hasegawa H., Murata J., Saiki I. (1997). *In vivo* antimetastatic action of ginseng protopanaxadiol saponins is based on their intestinal bacterial metabolites after oral administration. Oncol. Res..

[3] Hasegawa H., Lee K.S., Nagaoka T., Tezuka Y., Uchiyama M., Kadota S., Saiki I. (2000). Pharmacokinetics of ginsenoside deglycosylated by intestinal bacteria and its transformation to biologically active fatty acid esters. Biol. Pharm. Bull..

[4] Hasegawa H., Sung J.H., Benno Y. (1997). Role of human intestinal prevotella oris in hydrolyzing ginseng saponins. Planta Med..

[5] Akao T., Kida H., Kanaoka M., Hattori M., Kobashi K. (1998). Intestinal bacterial hydrolysis is required for the appearance of compound K in rat plasma after oral administration of ginsenoside Rb_1_ from *Panax ginseng*. J. Pharm. Pharmacol..

[6] Bae E.A., Park S.Y., Kim D.H. (2000). Constitutive beta-glucosidases hydrolyzing ginsenoside Rb_1_ and Rb_2_ from human intestinal bacteria. Biol. Pharm. Bull..

[7] Bae E., Choo M.K., Park E.K., Park S.Y., Shin H.Y., Kim D.H. (2002). Metabolism of ginsenoside R(C) by human intestinal bacteria and its related antiallergic activity. Biol. Pharm. Bull..

[8] Tawab M.A., Bahr U., Karas M., Wurglics M., Schubert-Zsilavecz M. (2003). Degradation of ginsenosides in humans after oral administration. Drug Metab. Dispos..

[9] Shin H.Y., Lee J.H., Lee J.Y., Han Y.O., Han M.J., Kim D.H. (2003). Purification and characterization of ginsenoside Ra-hydrolyzing *β*-D-xylosidase from bifidobacterium Breve K-110, a human intestinal anaerobic bacterium. Biol. Pharm. Bull..

[10] Hasegawa H., Sung J.H., Huh J.H. (1997). Ginseng intestinal bacterial metabolite IH901 as a new anti-metastatic agent. Arch. Pharm. Res..

[11] Hasegawa H. (2004). Proof of the mysterious efficacy of ginseng: Basic and clinical trials: Metabolic activation of ginsenoside: Deglycosylation by intestinal bacteria and esterification with fatty Acid. J. Pharmacol. Sci..

[12] Shin J.E., Park E.Y., Kim E.J.J., Hong Y.H., Lee K.T., Kim D.H. (2003). Cytotoxicity of compound K (IH-901) and ginsenoside Rh_2_, main biotransformatants of ginseng saponins by bifidobacteria, against some tumor cells. J. Ginseng Res..

[13] Ming Y., Chen Z., Chen L., Lin D., Tong Q., Zheng Z., Song G. (2011). Ginsenoside compound K attenuates metastatic growth of hepatocellular carcinoma, which is associated with the translocation of nuclear factor-κB p65 and reduction of matrix metalloproteinase-2/9. Planta Med..

[14] Jeong A., Lee H.J., Jeong S.J., Lee H.J., Lee E.O., Bae H., Kim S.H. (2010). Compound K inhibits basic fibroblast growth factor-induced angiogenesis via regulation of P38 mitogen activated protein kinase and AKT in human umbilical vein endothelial cells. Biol. Pharm. Bull..

[15] Park S., Lee H.J., Jeong S.J., Song H.S., Kim M., Lee H.J., Lee E.O., Kim D.H., Ahn K.S., Kim S.H. (2011). Inhibition of JAK1/STAT3 signaling mediates compound K-induced apoptosis in human multiple myeloma U266 cells. Food Chem. Toxicol..

[16] Cho S.H., Chung K.S., Choi J.H., Kim D.H., Lee K.T. (2009). Compound K, a metabolite of ginseng saponin, induces apoptosis via caspase-8-dependent pathway in HL-60 human leukemia cells. BMC Cancer.

[17] Wang C.-Z., Du G.-J., Zhang Z.Y., Wen X.-D., Calway T., Zhen Z., Musch M.W., Bissonnette M., Chang E.B, Yuan C.-S. (2012). Ginsenoside compound K, not Rb_1_, possesses potential chemopreventive activities in human colorectal cancer. Int. J. Oncol..

[18] Joh E.H., Lee I.A., Jung I.H., Kim D.H. (2011). Ginsenoside Rb_1_ and its metabolite compound K inhibit IRAK-1 activation--the key step of inflammation. Biochem. Pharmacol..

[19] Han G.C., Ko S.K., Sung J.H., Chung S.H. (2007). Compound K Enhances Insulin Secretion with Beneficial Metabolic Effects In Db/Db Mice. J. Agric. Food Chem..

[20] Li W., Zhang M., Gu J., Meng Z.J., Zhao L.C., Zheng Y.N., Chen L., Yang G.L. (2012). Hypoglycemic effect of protopanaxadiol-type ginsenosides and compound K on Type 2 diabetes mice induced by high-fat diet combining with streptozotocin via suppression of hepatic gluconeogenesis. Fitoterapia.

[21] Park J.S., Shin J.A., Jung J.S., Hyun J.W., Van L.T.K., Kim D.H., Park E.M., Kim H.S. (2012). Anti-inflammatory mechanism of compound K in activated microglia and its neuroprotective effect on experimental stroke in mice. J. Pharmacol. Exp. Ther..

[22] Zhou W., Yan Q., Li J.Y., Zhang X.C., Zhou P. (2008). Biotransformation of *Panax notoginseng* saponins into ginsenoside compound K production by Paecilomyces bainier sp. 229. J. Appl. Microbiol..

[23] Han Y., Sun B., Hu X., Zhang H., Jiang B., Spranger M.I., Zhao Y. (2007). Transformation of bioactive compounds by fusarium sacchari fungus isolated from the soil-cultivated ginseng. J. Agric. Food Chem..

[24] Yoo M.H., Yeom S.J., Park C.S., Lee K.W., Oh D.K. (2011). Production of aglycon protopanaxadiol via compound K by a thermostable *β*-glycosidase from *Pyrococcus furiosus*. Appl. Microbiol. Biotechnol..

[25] Chae S., Kang K.A., Chang W.Y., Kim M.J., Lee S.J., Lee Y.S., Kim H.S., Kim D.H., Hyun J.W. (2009). Effect of compound K, a metabolite of ginseng saponin, combined with gamma-ray radiation in human lung cancer cells *in vitro* and *in vivo*. J. Agric. Food Chem..

[26] Atopkin L.N., Denisenko V.A. (2006). Synthesis of 20(*S*)-protopanaxadiol 20-*O*-*β*-D-glucopyranoside, a metabolite of Panax ginseng glycosides, and compounds related to it. Chem. Nat. Compd..

[27] Hasegawa H., Suzuki R., Wakabayashi C., Murata J., Tezuka Y., Saiki I., Kadota S. (1998). Synthesis of a biologically active fluorescent derivative of GM1, a main Ginseng saponin metabolite formed by intestinal bacteria. Biol. Pharm. Bull..

[28] Lei J., Li X., Gong X.J., Zheng Y.N. (2007). Isolation, synthesis and structures of cytotoxic Ginsenoside derivatives. Molecules.

[29] Smith A., Nobmann P., Henehan G., Bourke P., Dunne J. (2008). Synthesis and antimicrobial evaluation of carbohydrate and polyhydroxylated non-carbohydrate fatty acid ester and ether derivatives. Carbohyd. Res..

[30] Kurahashi T., Mizutani T., Yoshida J.-I. (2002). Functionalized DMAP catalysts for regioselective acetylation of carbohydrates. Tetrahedron.

[31] Jansson P.-E., Kenne L., Schweda E. (1987). Nuclear magnetic resonance and conformational studies on monoacetylated methyl D-Gluco- and D-Galacto-pyranosides. J. Chem. Soc. Perkin Trans. I.

[32] Kawabata T., Muramatsu W., Nishio T., Shibata T., Schedel H. (2007). A catalytic one-step process for the chemo- and regioselective acylation of monosaccharides. J. Am. Chem. Soc..

